# Dynamic Mussel-Inspired Chitin Nanocomposite Hydrogels for Wearable Strain Sensors

**DOI:** 10.3390/polym12061416

**Published:** 2020-06-24

**Authors:** Pejman Heidarian, Abbas Z. Kouzani, Akif Kaynak, Ali Zolfagharian, Hossein Yousefi

**Affiliations:** 1 School of Engineering, Deakin University, Geelong, Victoria 3216, Australia; pheidarian@deakin.edu.au (P.H.); akif.kaynak@deakin.edu.au (A.K.); a.zolfagharian@deakin.edu.au (A.Z.); 2 Department of Wood Engineering and Technology, Gorgan University of Agricultural Sciences and Natural Resources, Gorgan 4913815739, Iran; hyousefi.ir@gmail.com

**Keywords:** dynamic hydrogels, tannic acid, chitin nanofibers, starch, self-healing, self-recovery

## Abstract

It is an ongoing challenge to fabricate an electroconductive and tough hydrogel with autonomous self-healing and self-recovery (SELF) for wearable strain sensors. Current electroconductive hydrogels often show a trade-off between static crosslinks for mechanical strength and dynamic crosslinks for SELF properties. In this work, a facile procedure was developed to synthesize a dynamic electroconductive hydrogel with excellent SELF and mechanical properties from starch/polyacrylic acid (St/PAA) by simply loading ferric ions (Fe^3+^) and tannic acid-coated chitin nanofibers (TA-ChNFs) into the hydrogel network. Based on our findings, the highest toughness was observed for the 1 wt.% TA-ChNF-reinforced hydrogel (1.43 MJ/m^3^), which is 10.5-fold higher than the unreinforced counterpart. Moreover, the 1 wt.% TA-ChNF-reinforced hydrogel showed the highest resistance against crack propagation and a 96.5% healing efficiency after 40 min. Therefore, it was chosen as the optimized hydrogel to pursue the remaining experiments. Due to its unique SELF performance, network stability, superior mechanical, and self-adhesiveness properties, this hydrogel demonstrates potential for applications in self-wearable strain sensors.

## 1. Introduction

Hydrogels are hydrophilic polymers cross-linked mostly by static covalent bonds in a three-dimensional (3D) structure [1,2,3,4]. They can maintain a large amount of water without losing their structures, and are suitable for many applications, including sensors [5], scaffolds [6], wound healing substrates [7], and actuators [8]. However, due to the presence of static bonds, they are usually prone to permanent failure while under load before any noticeable cracks appear, thus losing their functionality [1,2,9]. The insertion of dynamic non-covalent crosslinks within their networks can be considered as one feasible way to fabricate hydrogels with the ability to restore their structures and functionalities from damage, thus improving their safety, reliability, and durability. Furthermore, the reversibility of dynamic crosslinks in such hydrogels also imparts another interesting feature to their networks: autonomous self-healing and self-recovery (SELF) properties [1,3,10,11]. Therefore, dynamic hydrogels are good candidates for preparing soft flexible electronics, biomedicine, and wearable strain sensors [3,4,12,13]. However, the insertion of dynamic crosslinks may reduce the toughness of electroconductive hydrogels, the main prerequisite for fabricating hydrogel-based strain sensors that are subjected to repeated deformations [2,9]. Therefore, it is an ongoing challenge to fabricate an electroconductive hydrogel with toughness and autonomous SELF behaviors that are sufficiently suitable for wearable strain sensors because of the compromise between static crosslinks for mechanical strength and dynamic crosslinks for SELF properties [1,9,14,15].

The current solution to fabricate a tough SELF hydrogel relies on embedding dynamically modified nanofillers, e.g., nanoclay, graphene oxide, carbon nanotubes, and nanocellulose, within the network of hydrogels containing covalently cross-linked bonds [16,17,18]. By doing so, nanofillers with dynamic motifs increase the binding affinity at the interface of polymer chains and enhance the energy dissipation within the structure of hydrogels. However, due to employing irreversible covalent crosslinks, a full restoration of damaged hydrogels is not feasible. As an example, Shao et al. [18] employed tannic acid-coated cellulose nanocrystals (TA-CNCs) into a covalently cross-linked polyacrylic acid-aluminum-ion (PAA-Al^3+^) hydrogel to impart both SELF and mechanical strength to PAA hydrogel. Herein, TA, as a non-toxic, biocompatible plant-based polyphenol, provided strong metal-phenolic networks with Al^3+^ ions, thus imparting both SELF and mechanical strength to the PAA hydrogel; however, the presence of static bonds in the structure of the hydrogel restricted the fabrication of a fully reversible hydrogel [18].

In this work, TA-coated chitin nanofibers (TA-ChNFs) were employed as dynamic motifs for bestowing SELF and mechanical strength to a starch-based hydrogel without using any static bonds. ChNFs are highly crystallized fibrous structures that are mainly found in the exoskeleton of arthropods, e.g., crabs, shrimp, and insects [19,20]. They are formed linearly by the synthesis of glucosamine monomers connected by β-(1-4)-N-acetyl glucosamine linkages with an approximate diameter within a range of 2–20 nm [21]. ChNFs, similar to CNCs, have a good modifiability and provide excellent mechanical strength to hydrogels. Therefore, it is believed that TA-ChNFs can provide a high level of dynamic crosslinks between their adjacent nanofibers and polymer networks, thereby imparting both SELF and mechanical properties at the same time to a hydrogel network.

To fabricate hydrogels, polysaccharides are usually the most commonly used hydrophilic polymers because they are cheap, cytocompatible, biocompatible, and biodegradable, and among them, starch (St) is the most inexpensive and readily available polysaccharide [1]. In contrast with cellulose and chitin, which contain linear chains, St has highly-branched portions (amylopectin) with α(1,4)-anhydroglucose chains interlinked with α-(1 → 6)-glycosidic bonds, in association with some linear portions (amylose) with α(1,4)-linked anhydroglucose units [22]. As such, the presence of highly-branched chains in St always results in brittle and moisture-sensitive products with a poor mechanical strength. Therefore, it is almost impossible to use St in load-bearing applications, e.g., wearable strain sensors, due to its limited flexibility and stretchability. Herein, an electroconductive, tough hydrogel based on St with unique SELF properties was fabricated, suitable for wearable strain sensors.

Using such a hydrogel for wearable strain sensors also requires an external glue for fixing sensors onto substrates to prevent the interfacial delamination between the contacted substrates and sensors. This complicates the fabrication of hydrogels [23]. Therefore, there is a substantial need for developing self-adhesive soft wearable strain sensors. Polydopamine (PDA) is usually the main candidate for this additional feature, but its high cost and dark color may not always be useful for practical applications. In this regard, TA appears to be a better candidate than PDA because of its low-cost, non-toxic, nonirritant to human skin, and biocompatible plant-based nature [24,25]. Herein, by employing TA, a mussel-inspired self-adhesive performance was also added to the hydrogel. This resolves the need for using external glue to attach the hydrogel onto the contact substrate, thus avoiding interfacial delamination under a repeated deformation state and improving the stability of the signal detection.

## 2. Materials and Methods

### 2.1. Chemicals and Materials

Mechanically isolated chitin nanofibers (ChNFs) were supplied by Nano Novin Polymer Co. (Sari, Iran). Tannic acid (TA), acrylic acid (AA), starch (St), ammonium persulfate (APS), Tris buffer solution, and ferric chloride hexahydrate (FeCl_3_·6H_2_O) were purchased from Sigma-Aldrich (Castle Hill, Australia).

### 2.2. TA-ChNFs Preparation

The suspension of TA-ChNFs was prepared by a procedure proposed by Shao et al. [18]. In brief, the pH was first adjusted to 8.0 by dropwise adding Tris buffer solution into 150 cc of ChNFs suspension (∼1 wt.%). After that, 0.51 g of TA was loaded into the ChNFs suspension. The suspension was then magnetically stirred for 6 h at room temperature to coat the surface of ChNFs with TA. Next, the suspension was purified by a repeated centrifugation, followed by redispersing in distilled water. By doing so, the color of the suspension was changed from white to brown. The TA-ChNFs suspension was then sealed and cryopreserved at 4 °C.

### 2.3. Hydrogel Preparation

**Scheme 1:**
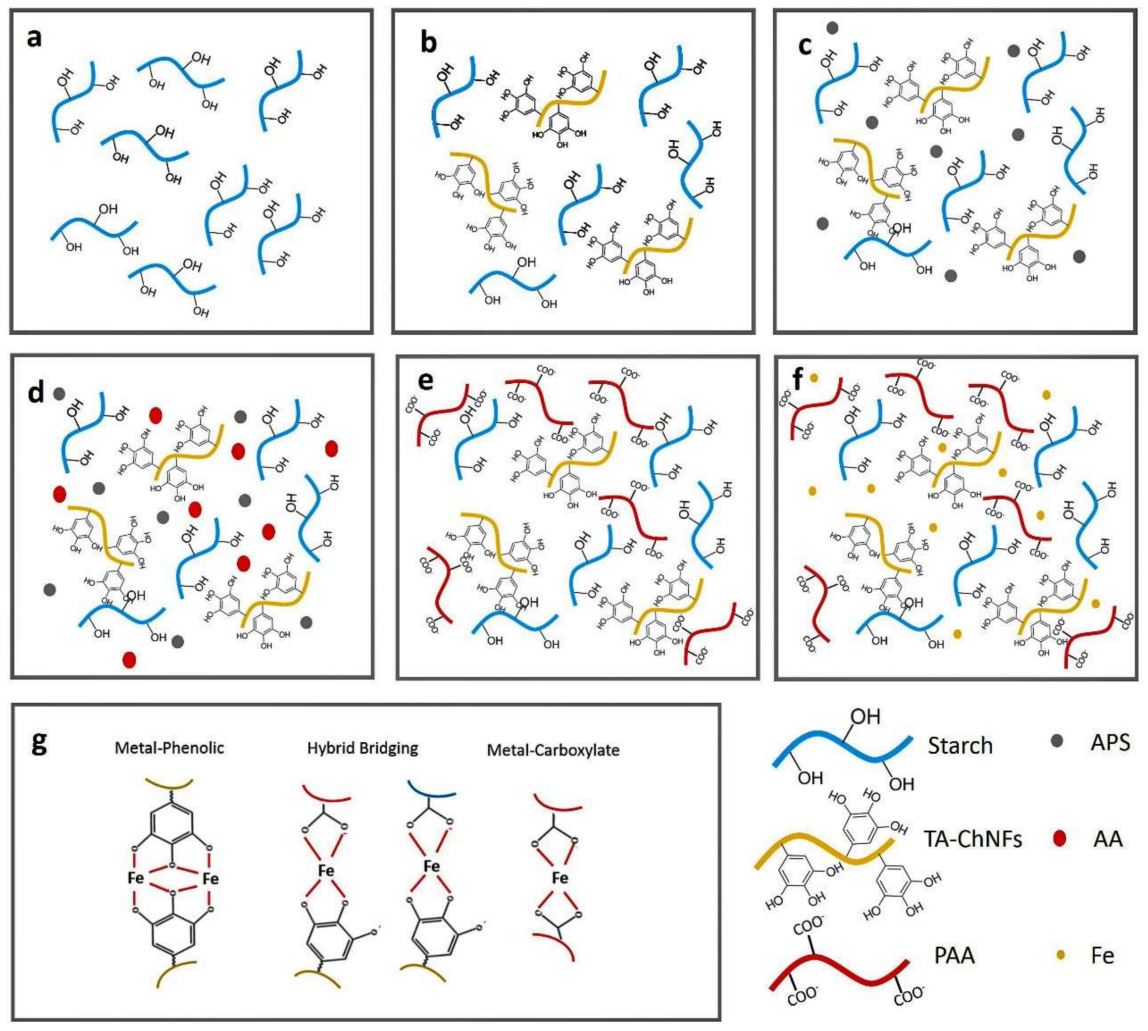
(**a**–**f**) Different steps for forming St/PAA/TA-ChNFs hydrogels; (**g**) different possible modes of coordination crosslinks.

The hydrogels were fabricated based on the method developed by Hussain et al. [26] for fabricating glycogen-PAA hydrogels. In brief, 2 g of St was dissolved in 24 cc distilled water at 80 °C for 2 h, followed by cooling it down to room temperature. Next, 50 mg APS was added into the dissolved St to activate the functional groups of St and stirred for 10 min. After that, 4 g of AA was added to the mixture and stirred for another 10 min. Finally, 0.1 M ferric (Fe^3+^) ions were loaded to the mixture, and the mixture was stirred again for 10 min at room temperature. TA-ChNF-loaded hydrogels were also fabricated at different concentrations (0.1, 0.5, 1, 1.5, and 2 wt.%) by loading TA-ChNFs into the dissolved St and sonicating for further 10 min in an ice-water bath to form a uniform mixture, followed by repeating the mentioned procedure for loading APS, AA, and Fe^3+^ ions. As seen in Scheme 1, after inclusion of TA-ChNF into St (Scheme 1b), AA monomers were polymerized using APS to impart a higher molecular polarity to starch/TA-ChNFs (Scheme 1c–e). Afterward, 0.1 M solution of Fe^3+^ ions was loaded into the St/PAA/TA-ChNFs mixture while mixing for almost 10 s at room temperature (Scheme 1f). Finally, superfluous cations from the Fe^3+^-loaded hydrogels were removed by soaking them in deionized water for 24 h. All samples were coded according to St/PAA/TA-ChNF (x%), which is the weight ratio of the TA-ChNF against AA/St (constant at 6 g). As an example, 1.0 wt.% TA-ChNF hydrogel refers to the hydrogel containing 60 mg of TA-ChNF and is coded as St/PAA/TA-ChNF (1%). Table 1 shows the detailed composition of the hydrogels, and Scheme 1g shows the possible coordination modes after formation of the hydrogel.

### 2.4. Hydrogel Characterization

The morphologies of ChNFs and TA-ChNFs were observed using transmission electron microscopy, TEM (JEOL, Tokyo, Japan, 2100). Each sample was diluted to about 0.001 wt.% with ethanol and sonicated well to separate the individual nanofibers and avoid aggregation during the analysis. Then, a quantity of 5 µL of the ChNFs was cast on perforated carbon-coated grids, and the excess ethanol was absorbed by a filter paper. An image analyzer program (version 1.52t, ImageJ, Bethesda, MD, USA) was used to measure the diameter of the nanofibers from the TEM images.

Next, the mechanical tests were performed using a universal mechanical tester equipped with a 200 N load cell at room temperature (Instron, Norwood, MA, USA) to find the hydrogel with the highest toughness at an optimum TA-ChNFs concentration. To do so, rectangular-shaped specimens, with dimensions of 10 mm in width, 6 mm in depth, and 35 mm in length, were prepared to test the mechanical properties of the TA-ChNF-loaded hydrogels at different concentrations (0.1, 0.5, 1, 1.5, and 2 wt.%). The constant stretching rate and initial distance between two clamps were respectively 60−160 mm/min and 15 mm. Prior to the test, the surface of hydrogels was coated with a layer of silicone oil to minimize water evaporation. The fracture strains of hydrogels were determined from the elongation at the break values. The toughness, that is the ability of the hydrogel to absorb energy prior to fracture, was calculated from the area under the stress–strain curves. All tensile tests were repeated five times. Rheological measurements were also performed to support the mechanical characterization using a rotational rheometer (TA Instruments, New castle, Delaware, USA) equipped with a parallel plate geometry (40 mm in diameter and a gap of 49 μm) at different strains and a frequency of 1.0 Hz.

After determining the hydrogel with the highest toughness, further experiments were performed on the samples with the optimized TA-ChNFs concentration. Scanning electron microscopy, SEM (Zeiss, Supra, Oberkochen, Germany), was employed to investigate the morphology of the freeze-dried hydrogel before and after loading TA-ChNFs. To do so, the optimized hydrogel was cut to expose the inner structure and coated with gold in a vacuum coater to avoid charging and then observed under SEM operating at 8.5 and 17.5 kV. The spectra of ChNFs, TA-ChNFs, and the hydrogels at the optimized TA-ChNFs concentration were obtained using FTIR (Bruker Vertex 70, Billerica, MA, USA) between 600–4000 cm^−1^. Prior to the test, the samples were dried in a vacuum oven for 24 h.

The healing efficiency of hydrogels was also calculated according to the strength ratio between the healed hydrogel and the original one at different TA-ChNFs concentrations (0.1, 0.5, 1, 1.5, and 2 wt.%). Furthermore, a visual inspection was conducted to evaluate the self-healing properties of the hydrogel at an optimum TA-ChNFs concentration by cutting the hydrogel into pieces, followed by immediately recombining the pieces into the original shape through self-adhesion.

The self-adhesiveness properties of the hydrogel were measured at an optimum TA-ChNFs concentration on different substrates (plastic, glass, metal, leather, and rubber with 25 × 100 × 1 mm^3^ dimensions) using the same universal mechanical tester under ambient conditions. For this purpose, all solid specimens were washed with water and ethanol and dried to remove any dirt from their surfaces. Next, the hydrogels (with 20 × 20 × 1 mm^3^ dimensions) were attached to the substrates and were pulled at a crosshead speed of 10 mm/min until separation. Each sample for measuring the self-adhesive test was repeated five times [27].

## 3. Results and Discussion

### 3.1. Tannic Acid Coated-Chitin Nanofiber-Assisted Hydrogels

**Figure 1:**
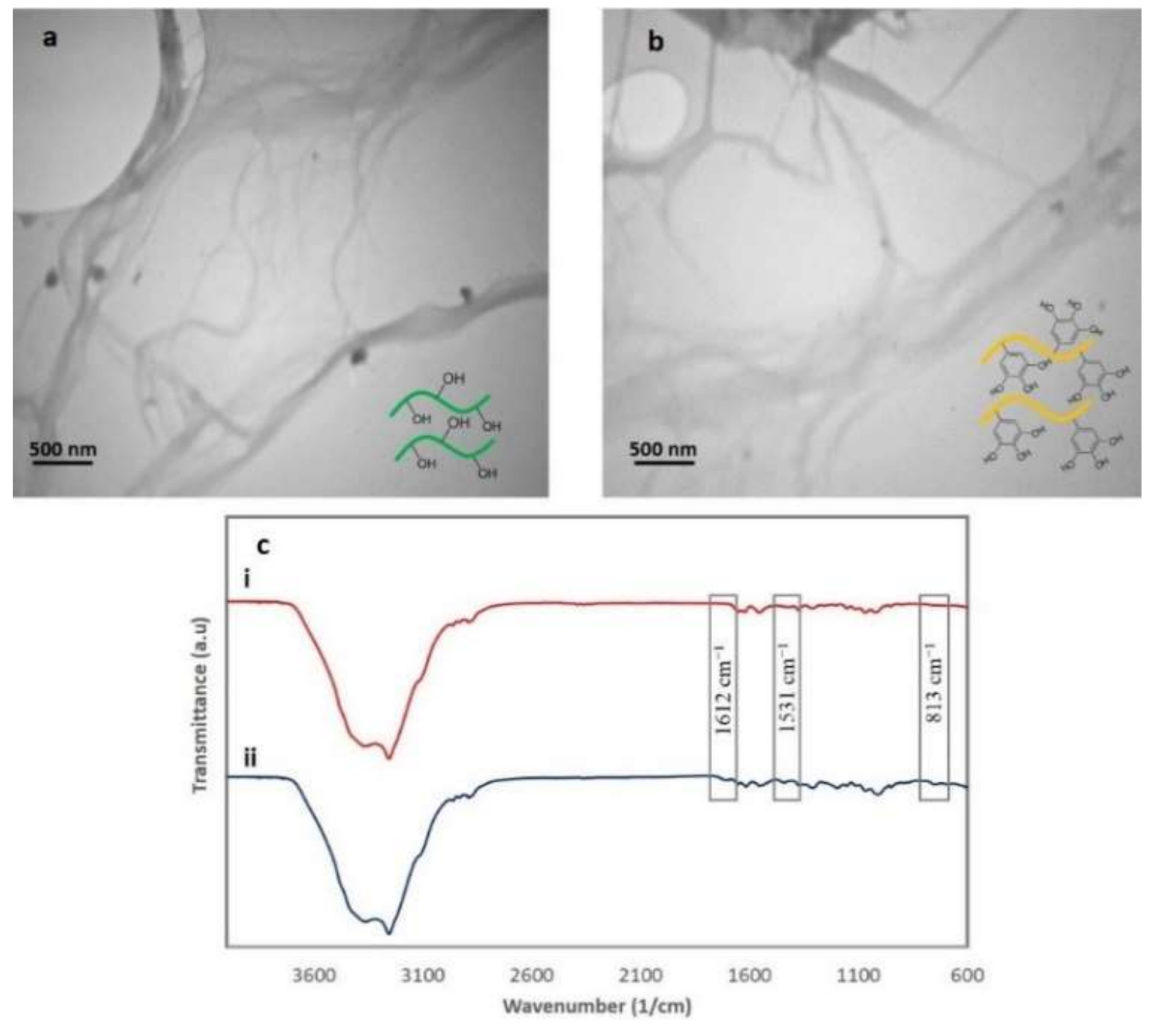
TEM image of (**a**) ChNFs and (**b**) TA-ChNFs; and FTIR results of (**ci**) ChNFs and (**cii**) TA-ChNFs.

According to the TEM image of ChNFs (Figure 1a), the average diameter of ChNFs is in the nanosize range (48 ± 12 nm), while their lengths are in the micrometer scale. Therefore, ChNFs have a high aspect ratio containing a lot of hydroxyl groups able to interact with the pyrogallol/catechol groups of TA. Based on the TEM image (Figure 1b), the average diameter of TA-ChNFs is slightly thicker than ChNFs (51 ± 11 nm), which may be due to the deposition of TA onto ChNFs. The successful deposition of TA on ChNFs was confirmed using the FTIR test. As shown in Figure 1c, unlike the pristine ChNFs (Figure 1ci), there is a detectable peak at 813 cm^−1^ in the TA-ChNFs spectrum (Figure 1cii) due to the introduction of C=C in benzene rings. There are also detectable peaks at 1531 and 1612 cm^−1^ due to stretching vibrations of C−C aromatic groups in the spectrum of TA-ChNFs. These results are in agreement with the results obtained by Shao et al. [18] when depositing TA on the surfaces of cellulose nanocrystals.

Inspired by the adhesiveness of blue mussels, a tough hydrogel with SELF properties was fabricated via the incorporation of TA-ChNFs into St/PAA, followed by loading Fe^3+^ ions. The nearly instant gelation of 0.5 wt.% TA-ChNF-reinforced St/PAA is shown in Video S1 from Supplementary Materials. The instant gelation was not observed for the mixture containing 0.1 wt.% TA-ChNFs (Video S2). The instant gelation can be attributed to the sufficient contribution of pyrogallol/catechol groups of TA performing intermolecular interactions with Fe^3+^ ions, thus forming coordination crosslinks. In fact, after loading Fe^3+^ ions into the TA-ChNFs-assisted mixture, three possible coordination modes are likely to happen, consisting of hybrid bridging, metal-carboxylate coordination, and metal-phenolic coordination (Scheme 1g). All of these modes, as well as the presence of hydrogen bonding, result in a reversible dynamic network that can instantly form a 3D gel.

### 3.2. Mechanical and Rheological Properties

**Figure 2:**
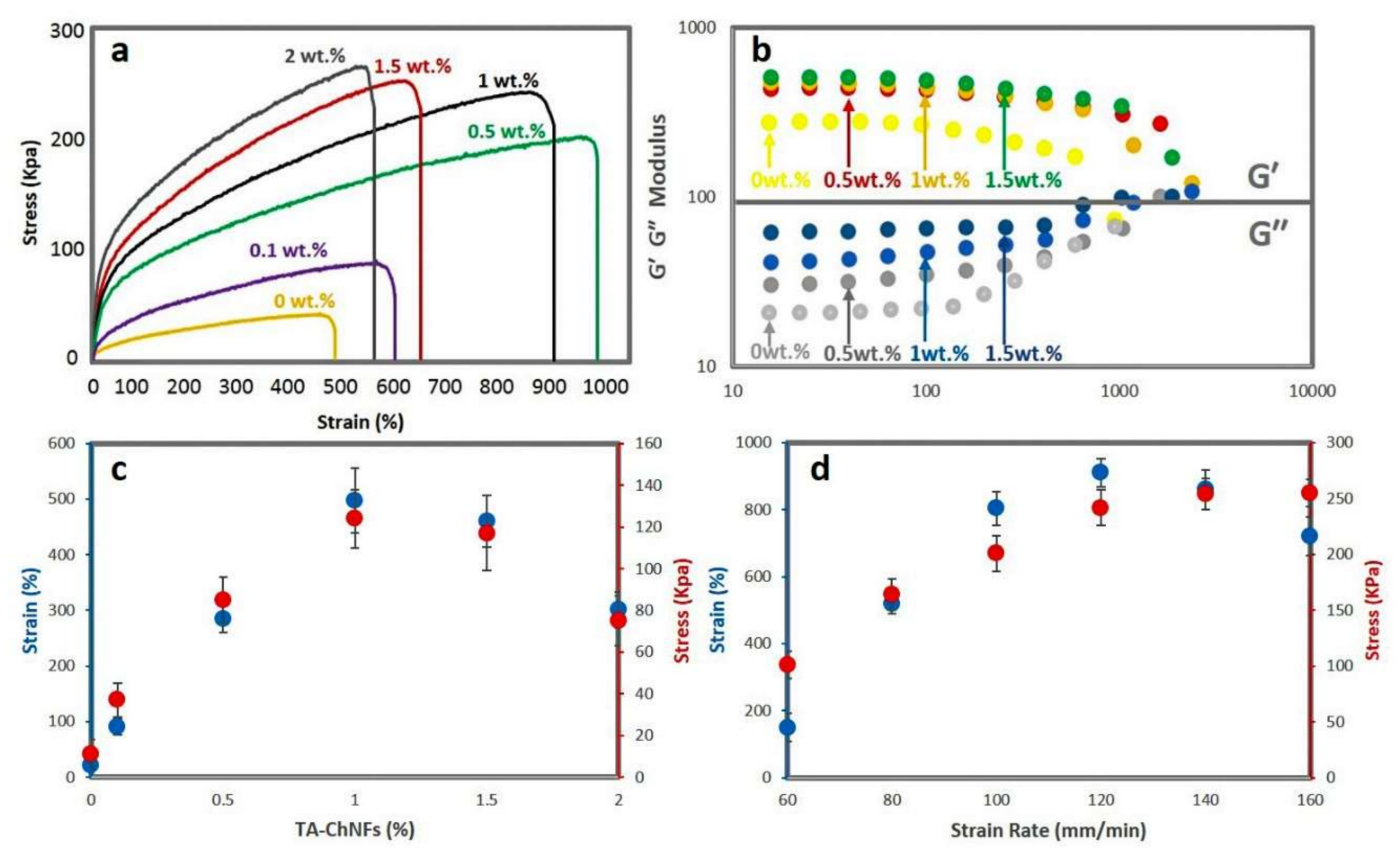
(**a**) Tensile stress–strain curves of PAA/St hydrogels at different TA-ChNFs concentrations; (**b**) Storage modulus and loss modulus of the hydrogel at different TA-ChNFs percentages; (**c**) tensile properties for notched hydrogels at different TA-ChNFs concentrations; (**d**) tensile properties of the hydrogel reinforced by 1 wt.% TA-ChNFs at different strain rates.

Since toughness is one of the most important prerequisites of a wearable strain sensor, mechanical measurements were first performed on all hydrogels at different TA-ChNFs concentrations (0.1, 0.5, 1, 1.5, and 2 wt.%) to indicate the hydrogel with the highest toughness. As mentioned, St is inherently a brittle polymer, so the graft polymerization of vinyl monomers, e.g., acrylic acid and acrylamide, can be a facile way to modify the mechanical strength of St. Moreover, the incorporation of nanofillers within the St network can improve the mechanical strength of St [28]. Therefore, it is anticipated that the incorporation of TA-ChNFs and the graft polymerization of AA without using any static cross-linkers can considerably increase the toughness of St-based hydrogels while imparting excellent SELF properties to them. These two properties (toughness and SELF properties) usually oppose each other [9]. Figure 2a displays the stress–strain curves of the hydrogels incorporated with different concentrations of TA-ChNFs (0.1, 0.5, 1, 1.5, and 2 wt.%). As can be seen, the incorporation of TA-ChNFs as both dynamic motifs and nanofillers significantly improved the mechanical properties of the St-based hydrogels. The highest stretchability of 1015% is observed for the 0.5 wt.% TA-ChNF-reinforced hydrogel. The highest tensile strength is seen for the 2 wt.% TA-ChNF-reinforced hydrogel (274.8 kPa). Moreover, the 1 wt.% TA-ChNF-reinforced hydrogel shows the highest toughness (1.43 MJ/m^3^), which is 10.5-fold higher than the unreinforced hydrogel, whereas the decrease in toughness at higher TA-ChNFs may be due to a higher degree of crosslinking at higher TA-ChNFs concentrations.

To evaluate the effects of different percentages of TA-ChNFs on the mechanical properties of the hydrogels, rheological measurements were performed. As seen in Figure 2b, the storage modulus (G’) of the hydrogel is higher than its loss modulus (G″), and the inclusion of TA-ChNFs, even at a very low concentration (0.5 wt.%), increases both the storage modulus (G′) and the loss modulus (G″) of the hydrogel; however, the trend is approximately the same at different TA-ChNFs concentrations, mainly due to both the nano-reinforcing and dynamic cross-linking effects of TA-ChNFs via non-covalent interactions. To assess the notch sensitivity of the TA-ChNF-reinforced hydrogels at different concentrations, all hydrogels were notched and stretched under tensile loading. It was observed that the hydrogel reinforced by 1 wt.% TA-ChNF had the highest notch-insensitivity, remaining remarkably stable and blunted. The toughness behavior of this hydrogel can be considered as the main reason for the resistance of hydrogel against crack propagation (Figure 2c). Based on the results from the mechanical, rheological, and notch-insensitivity measurements, the 1 wt.% TA-ChNF-reinforced hydrogel was considered as the optimum sample to pursue the remaining experiments.

The mechanical properties of the 1 wt.% TA-ChNF-reinforced hydrogel at different stretching rates (60–160 mm/min) was then tested to track the strain-rate dependency of the hydrogel. It was observed that the higher strain rates resulted in a higher breaking stress, reaching a maximum at 140 mm/min, beyond which the strain rate dependency of the tensile strength diminished. This can be attributed to a reduced dissipation energy by the coordination bonds [18]. While increasing the stretching rate up to 120 mm/min increased the breaking strain of the hydrogel, it decreased after 120 mm/min, mainly because of the inability of the broken physical bonds to reform (Figure 2d).

### 3.3. Morphological and FTIR Studies

**Figure 3:**
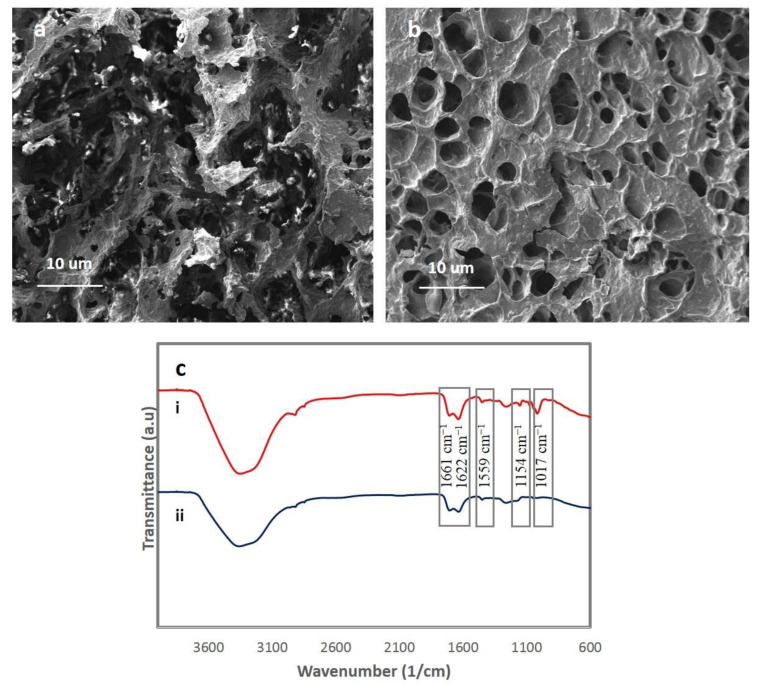
SEM images of (**a**) the unreinforced hydrogel and (**b**) the reinforced hydrogels with 1 wt.% TA-ChNFs; FTIR results of (**ci**) 1 wt.% TA-ChNF-reinforced hydrogels and (**cii**) unreinforced hydrogels.

In the next step, the morphological and FTIR studies of the optimized hydrogel were performed. Figure 3 shows the fracture surface of the pristine and 1 wt.% TA-ChNF-reinforced hydrogels. As can be seen, there exists a significant difference in the pore size and morphology of the reinforced and unreinforced hydrogels. While the reinforced hydrogel with 1 wt.% TA-ChNFs (Figure 3b) has a smaller, denser, and more uniform pore size, with a larger surface area, its unreinforced counterpart (Figure 3a) lacks any homogeneity and uniformity in pore size distribution. Therefore, it can be stated that the presence of TA-ChNFs enhances the homogeneity of the network by providing reversible interactions with the matrix. It can also be noted that the uniform structure of the 1 wt.% TA-ChNF-reinforced hydrogel resulted in better mechanical properties compared to its unreinforced counterpart.

Based on the FTIR results for the 1 wt.% TA-ChNF-reinforced hydrogel, there exist two amide I bands at 1622 and 1661 cm^−1^ and the amide II band at 1559 cm^−1^ (Figure 3ci,cii) due to the graft polymerization of AA on starch, and two characteristic bands at 1154 and 1017 cm^−1^ in Figure 3ci after the inclusion of 1 wt.% TA-ChNFs into the system. According to Ifuku et al. [29], these peaks are the C–O and C=O stretching vibration modes of the carboxylic acid resulting from the grafting of AA onto the chitin nanofibers.

### 3.4. Self-Healing and Self-Recovery (SELF) Properties

**Figure 4:**
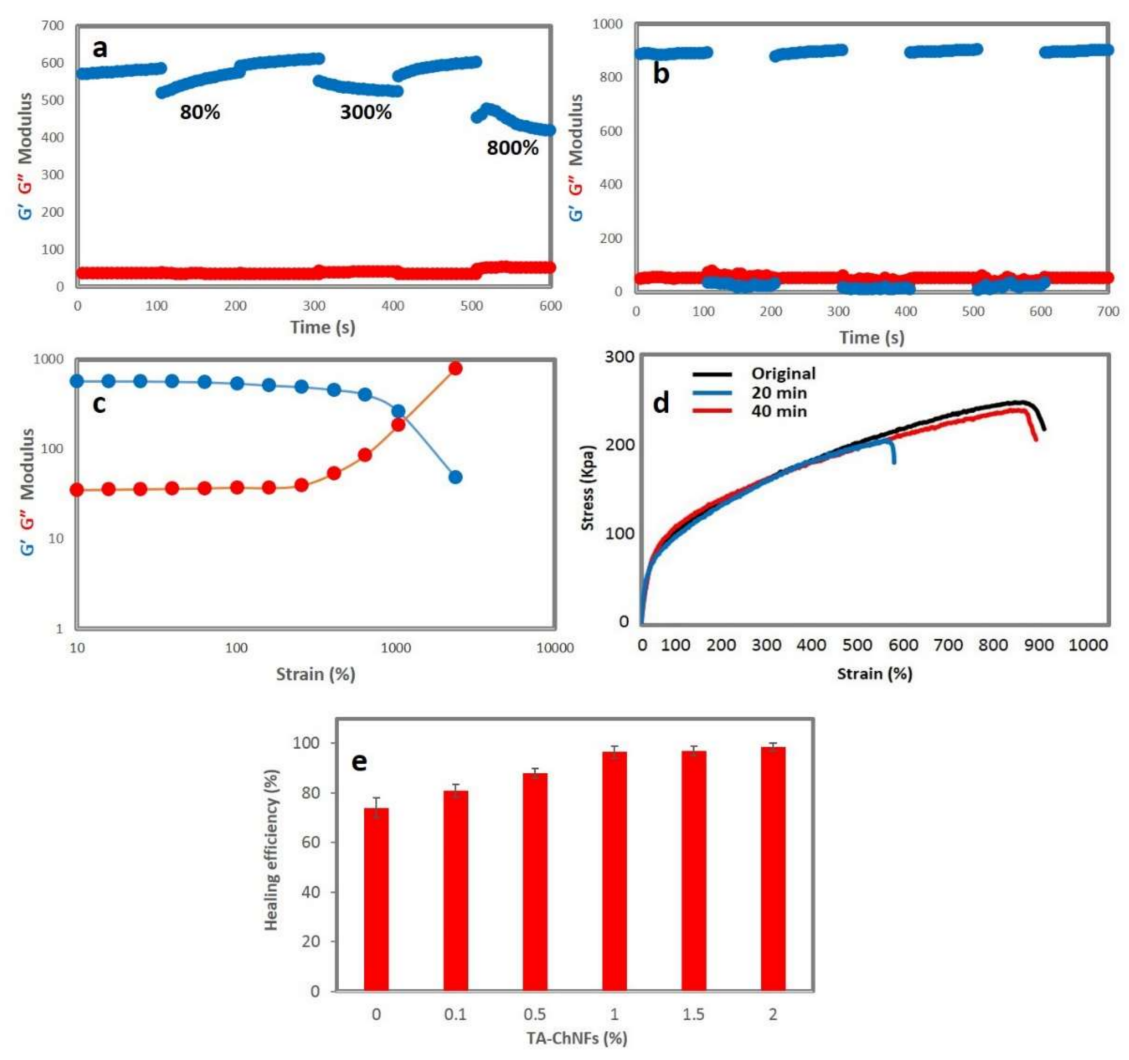
(**a**) Breakup and reformation ability of the hydrogel containing 1 wt.% TA-ChNFs at different strains = 1, 80, 300, and 800% and a fixed time interval; (**b**) breakup and reformation ability of the hydrogel containing 1 wt.% TA-ChNFs at 1 and 1000% strains and a fixed time interval; (**c**) shear-thinning behavior of the hydrogel containing 1 wt.% TA-ChNFs; (**d**) typical stress–strain curves of the pristine and healed hydrogels containing 1 wt.% TA-ChNFs; and (**e**) healing efficiency of the hydrogels at different TA-ChNFs.

Using the rheological measurement, the self-recovery of the hydrogel containing 1 wt.% TA-ChNFs was demonstrated at the microscopic level. For this purpose, an alternate step strain test (strain = 1, 80, 300, 800, and 1000%) at a fixed time interval (100 s) was performed (Figure 4a,b). As can be seen, under small oscillatory strains, the hydrogel network is intact thanks to the presence of TA-ChNFs as dynamic motifs. It can be said that the polymer chains in the hydrogel are attached by TA-ChNFs; thus, they can bear a large deformation and show a rapid and complete self-recovery at a low strain of 1% due to the interactions between the nanofillers and matrix. This proves the excellent self-recovery of the hydrogel under repeatable cycles and the fact that the hydrogel could withstand large deformations without showing shear-thinning behavior, which stands in contrast with many self-healing hydrogels due to the presence of strong dynamic motifs created by TA-ChNFs [9,30,31]. At a 1000% strain, the value of G′ and G″ overlapped, which means the structure of the polymer hydrogel starts collapsing at such large strains (Figure 4b). When stepping the strain back to 1% at a fixed frequency (1.0 Hz), an instantaneous self-recovery to the gel-like character (G’ > G″) was observed in each repeatable cycle of the recovery. This observation can be attributed to a fully dynamic network that enables a response to the strain at a molecular level. The hydrogel also showed a shear-thinning behavior at strains higher than 1000%, which indicates the transformation of the hydrogel into its sol-gel state due to the destruction of the hydrogel network (Figure 4c).

To evaluate the self-healing performance of the hydrogel, a macroscopic test was performed using a direct visual inspection, followed by a tensile test. As shown in Video S3, a cylindrical-shaped hydrogel containing 1 wt.%. TA-ChNFs was cut into several pieces, and the pieces were then immediately rejoined together without applying any pressure. As seen, the hydrogel instantly healed itself without any external stimuli or healing agents. Moreover, the self-healed hydrogel showed immediate and stable self-support and stretchability across the cutlines due to the strong dynamic crosslinks. Clearly, the hydrogel showed a good healing ability, stability, and mechanical properties due to the presence of the rigid but dynamic TA-ChNFs motifs together with the coordination bonds.

To further investigate the effect of time on the healing efficiency of hydrogels, further tensile tests were performed by defining the healing efficiency as a tensile strength ratio between the healed and original hydrogel at the breaking point. As shown in Figure 4d, the hydrogel shows a time-dependent self-healing performance, and the tensile strength increases significantly by increasing the time, especially during the earlier step of healing. The self-healing efficiency reached 80% after 20 min, but after that it slowed down slightly and reached 96.5% after 40 min. Therefore, it can be said that this rapid and autonomous self-healing performance of the hydrogel is due to the presence of coordination crosslinks and TA-ChNFs dynamic motifs. Figure 4e shows the influence of different TA-ChNFs concentrations on the healing efficiency of the hydrogel. As can be seen, by adding 2 wt.% TA-ChNFs, the healing efficiency reached 98.5% after 40 min of healing. Therefore, the presence of TA-ChNFs plays a crucial role in the high SELF performance, network stability, and superior mechanical properties of the hydrogel due to providing dynamic metal-phenol networks.

### 3.5. Self-Adhesiveness Properties

**Figure 5:**
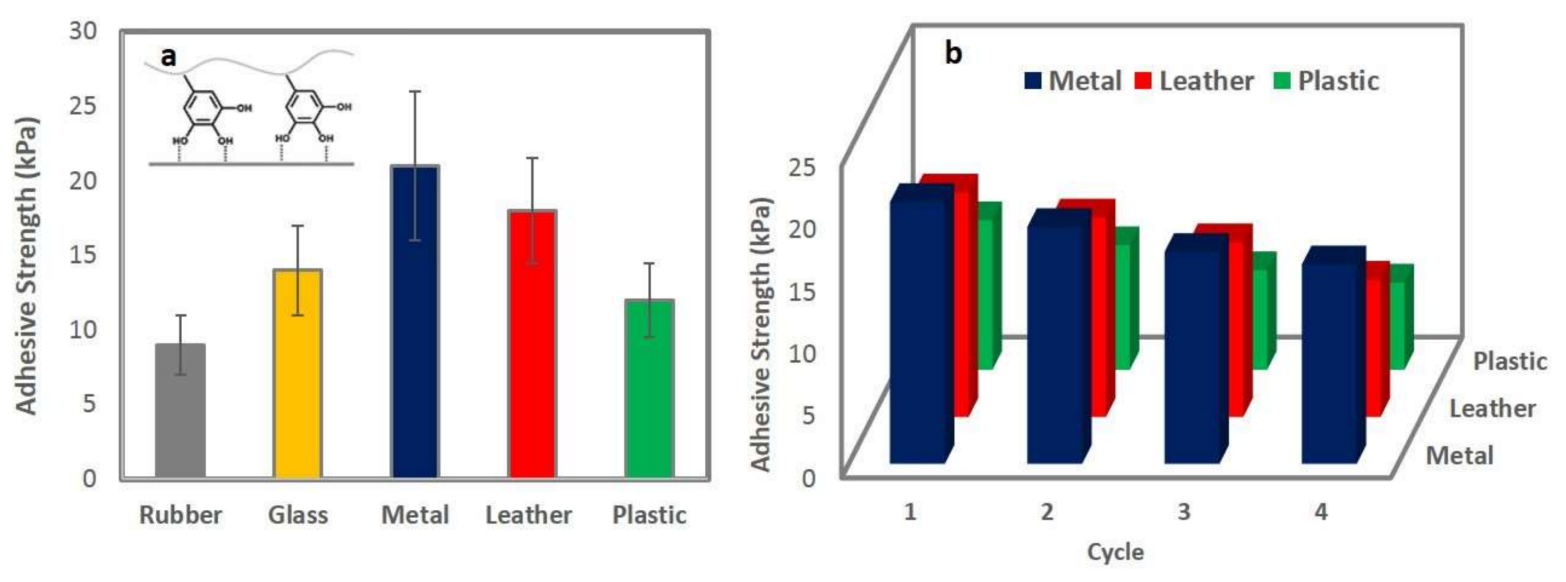
(**a**) Self-adhesive strength of the 1 wt.% TA-ChNF-reinforced hydrogel on different substrates measured by a tensile test; (**b**) cyclic self-adhesive tests on different substrates.

The incorporation of TA into ChNFs, in addition to providing high SELF and mechanical properties, can impart a mussel-inspired adhesive mechanism to the hydrogels due to the presence of pyrogallol/catechol groups in TA, allowing the hydrogel to adhere to almost any surface [18,23]. As demonstrated in Video S4, the hydrogel containing 1 wt.% TA-ChNFs can merge the glass–plastic slides while lifting a load of 5 kg without using extra glue. It has been reported that the mussel-inspired adhesive mechanism of hydrogels depends on the availability of pyrogallol/catechol moieties and the type of interactions at the hydrogel–substrate interface, while the mechanical strength of the hydrogel depends on the number of TA/Fe^3+^ coordinations [32,33]. The self-adhesive properties of the hydrogel reinforced by 1 wt.% TA-ChNFs were quantified to different surfaces consisting of rubber, metal, glass, plastic, and leather using a tensile test. As Figure 5a depicts, the highest self-adhesive strength was found between hydrogel–metal interfaces. This can be attributed to the presence of metal complexation and hydrogen bonding at the interfacial layer [23]. The adhesiveness of hydrogel–plastic can be attributed to hydrophobic interactions, e.g., π-π stacking or CH-π interaction. Hydrogen bonding can also be considered for the adhesiveness of the hydrogel to rubber, glass, and leather. [18] A cyclic peel-off test was also performed four times by adhering 1 wt.% TA-ChNF-reinforced hydrogel onto different substrates and peeling them off by the tensile load, followed by re-adhering them onto the same substrates and repeating the test (Figure 5b). Based on the results, the hydrogel showed good repeatable self-adhesive properties. As an example, 76% of the initial self-adhesive strength between the hydrogel–metal at the first cycle was restorable after the fourth cycle. As Video S5 shows, the hydrogel can easily adhere to rubbery gloves while being stretched without adding any additional adhesive tapes. Therefore, the added functionality of self-adhesiveness to the hydrogel can, without influencing the mechanical performance and self-healing, increase the versatility of the hydrogel in many practical applications, e.g., wearable strain sensors [32].

### 3.6. Electrical Conductivity and a Potential Application

**Figure 6:**
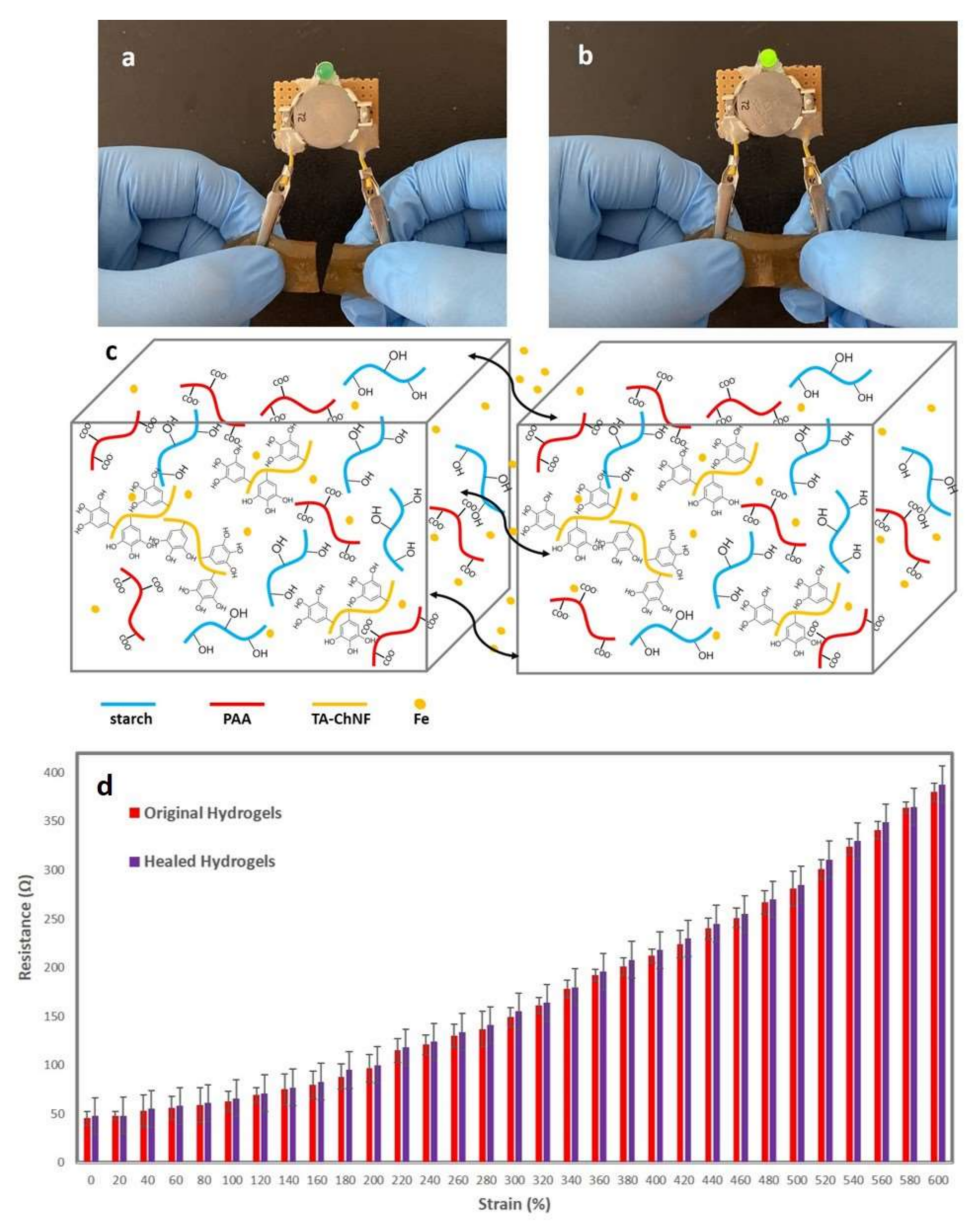
(**a**) Before connecting the cut hydrogel containing 1 wt.% TA-ChNFs; (**b**) after connecting the cut hydrogel with 1 wt.% TA-ChNFs; (**c**) the mechanism of electrical self-healing of the hydrogel and (**d**) the resistance of the hydrogel at different strains.

The electrical conductivity is demonstrated by a light-emitting diode (LED) indicator and the hydrogel reinforced by 1 wt.% TA-ChNFs as the conductor in Videos S6 and S7. As shown in Video S6, the LED bulb reversibly darkened and lit up by applying and releasing the stress due to the presence of local disconnections, indicating the fast resistance response of the hydrogel to the strain. The soft electrical switching behavior of the hydrogel was observed using the LED indicator and is shown in Video S7. As can be seen, the LED bulb in the electric circuit was lit by connecting the hydrogel to the electric circuit. The hydrogel was then cut in half using scissors, and the cut pieces were again connected. As seen in Figure 6a,b, The LED bulb in the electric circuit was instantly lit, and the circuit was restored due to the migration of free Fe^3+^ ions from one side to another (Figure 6c), imparting a restorable ionic conductivity to the hydrogel. The resistance of the hydrogel was measured using a multimeter at a probe distance of 1 cm. It was observed that by increasing the strain values from 0 to 600%, the resistance of the hydrogel increased. This can be attributed to the increased distance between the conductive segments of the hydrogel network, resulting in increased local disconnections within the network (Figure 6d) [23].

**Figure 7:**
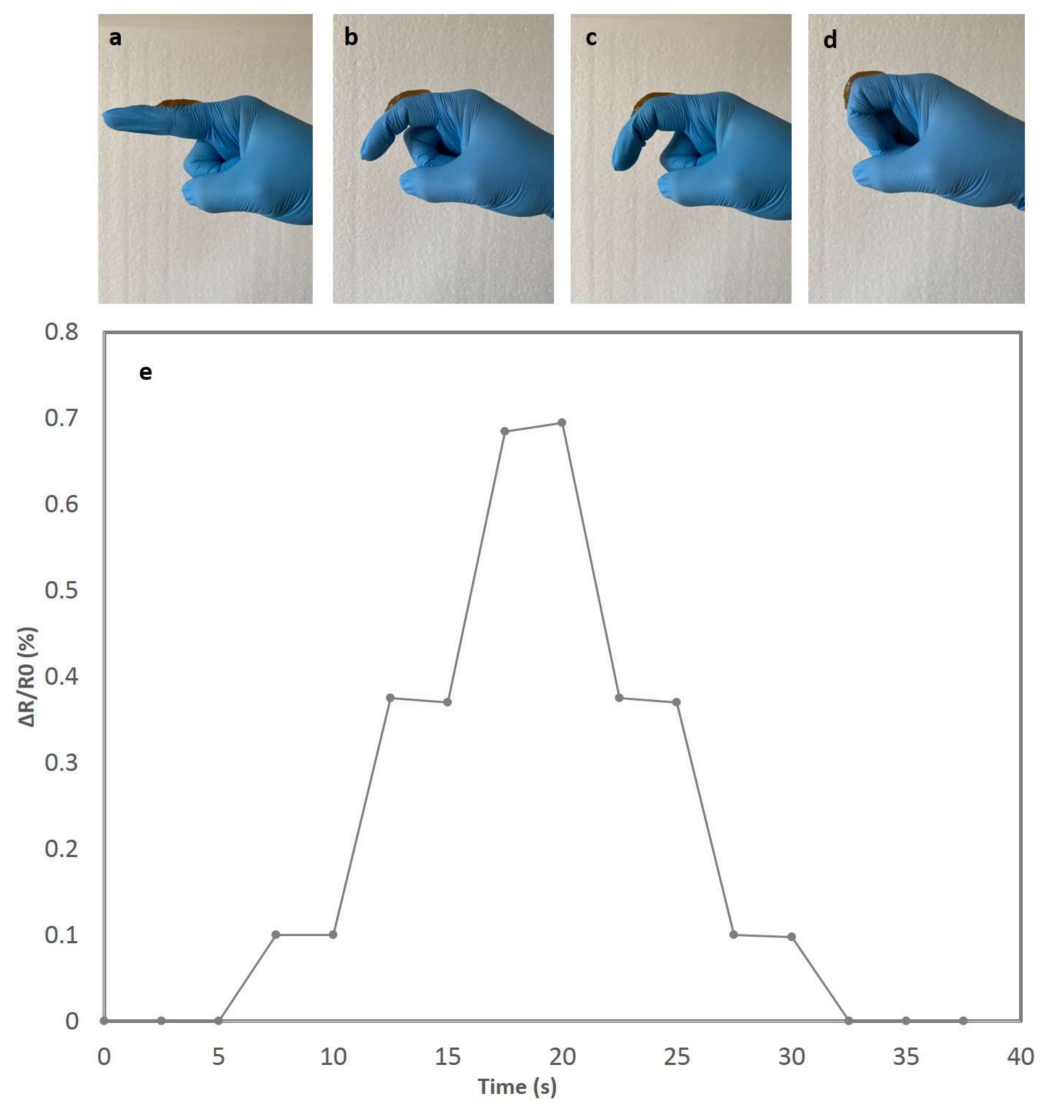
(**a–d**) Adhered 1 wt.% TA-ChNFs hydrogel to the first author’s forefinger while bending to approximate angles and (**e**) its relative resistance change in real time.

Most hydrogel-based wearable sensors require an external glue for affixing to the body. Moreover, they are usually brittle and are likely to lose their functionality while under load [18]. By taking advantage of TA-ChNFs, a self-adhesive hydrogel with the potential to solve this problem can be fabricated. The potential use of the hydrogel as a self-wearable strain sensor was examined by adhering it onto the top surface of the forefinger without employing any glue for detecting the bending movement of the finger. As Figure 7 depicts, the hydrogel could be easily attached to the forefinger without using any glue, and by increasing the angle of bending from 0° to approximately 90° the resistance of the hydrogel increased, whereas the resistance did not change when the forefinger was held static at approximately the same angle. Interestingly, by returning the bending angle to 0°, the initial resistance of the hydrogel was restored. Hence, the hydrogel can distinguish between different bending angles based on the relative resistance changes in real time, making our hydrogel an ideal candidate for self-wearable strain sensors.

## 4. Conclusions

In this work, a dynamic mussel-inspired hydrogel was designed and fabricated by incorporating Fe^3+^ ions and TA-ChNFs into a starch-based network. TA-ChNFs play the role of nanofillers and dynamic cross-linkers, thus imparting a SELF ability and high strength to the hydrogel. The hydrogel shows a high SELF ability, mechanical strength, and electro-conductivity. According to our results, the hydrogel reached the highest toughness and notch insensitivity after loading 1 wt.% TA-ChNFs into the hydrogel. Moreover, the hydrogel reinforced by 2 wt.% TA-ChNFs showed the highest self-healing efficiency (98.5%) after 40 min. Additionally, the hydrogel showed repeatable self-adhesive properties, with the ability to attach to almost any surface because of the existence of pyrogallol/catechol groups in TA. The hydrogel is also able to monitor and distinguish motions, showing a good capability as a soft wearable strain sensor. Thus far, there has not been any available research on using TA-ChNFs and starch as potential candidates for fabricating such hydrogels. We anticipate that the hydrogel will be ideally suited for use in self-wearable flexible strain sensors because of its excellent self-healing ability—without needing any external stimuli—and reliable mechanical, electrical, and self-adhesive properties.

## Figures and Tables

**Table 1:** Compositions of the hydrogels.

Code	TA-ChNFs (wt.%)	TA-ChNFs (mg)	AA (g)	St (g)	APS (mg)	Water (cc)
St/PAA	0	0	4	2	50	24
St/PAA/TA-ChNF (0.1%)	0.1	6	4	2	50	24
St/PAA/TA-ChNF (0.5%)	0.5	30	4	2	50	24
St/PAA/TA-ChNF (1%)	1	60	4	2	50	24
St/PAA/TA-ChNF (1.5%)	1.5	90	4	2	50	24
St/PAA/TA-ChNF (2%)	2	120	4	2	50	24

## Supplementary Materials

The following are available online at https://www.mdpi.com/2073-4360/12/6/1416/s1,. Video S1: 0.5 wt.% TA-ChNF-reinforced hydrogel. Video S2: 0.1 wt.% TA-ChNF-reinforced hydrogel. Video S3: Visual self-healing inspection of a cylindrical-shaped 1 wt.%. TA-ChNF-reinforced hydrogel. Video S4: Merging glass-plastic slides by a 1 wt.%.TA-ChNF-reinforced hydrogel. Video S5: 1 wt.%.TA-ChNF-reinforced hydrogel adhered to rubbery gloves while being stretched without adding any additional adhesive tapes. Video S6: Darkening and lightening of 1 wt.%.TA-ChNF-reinforced hydrogel after applying and releasing the stress using an LED bulb indicator. Video S7: Electrical self-healing ability of the hydrogel reinforced by 1 wt.%.TA-ChNF.
